# Longitudinal temperature measurement can determine humane endpoints in BALB/c mouse models of ESKAPEE infection

**DOI:** 10.1080/21505594.2023.2186331

**Published:** 2023-03-28

**Authors:** Randal Scott Dudis, Ting Y. Wong, Mariel G. Escatte, Yonas A. Alamneh, Rania Abu-Taleb, Wanwen Su, Christine Czintos, Timothy A. Fitzgerald, Yoann Le Breton, Daniel V. Zurawski

**Affiliations:** a Veterinary Services Program, Center for Enabling Capabilities, Walter Reed Army Institute of Research, Silver Spring, MD, USA; b Wound Infections Department, Bacterial Diseases Branch, Center for Infectious Diseases Research, Walter Reed Army Institute of Research, Silver Spring, MD, USA

**Keywords:** Temperature, ESKAPEE, murine pulmonary infection model, BALB/c, humane endpoints, replacement, reduction, refinement (3Rs)

## Abstract

Antimicrobial resistance (AMR) is a worldwide problem, which is driving more preclinical research to find new treatments and countermeasures for drug-resistant bacteria. However, translational models in the preclinical space have remained static for years. To improve animal use ethical considerations, we assessed novel methods to evaluate survival after lethal infection with ESKAPEE pathogens (*Enterococcus faecium, Staphylococcus aureus*, *Klebsiella pneumoniae*, *Acinetobacter baumannii*, *Pseudomonas aeruginosa*, *Enterobacter cloacae*, and *Escherichia coli*) in pulmonary models of infection. Consistent with published lung infection models often used for novel antimicrobial development, BALB/c mice were immunosuppressed with cyclophosphamide and inoculated intranasally with individual ESKAPEE pathogens or sterile saline. Observations were recorded at frequent intervals to determine predictive thresholds for humane endpoint decision-making. Internal temperature was measured via implanted IPTT300 microchips, and external temperature was measured using a non-contact, infrared thermometer. Additionally, clinical scores were evaluated based on animal appearance, behaviour, hydration status, respiration, and body weight. Internal temperature differences between survivors and non-survivors were statistically significant for *E. faecium, S. aureus*, *K. pneumoniae*, *A. baumannii*, *E. cloacae*, and *E. coli*, and external temperature differences were statistically significant for *S. aureus*, *K. pneumoniae*, *E. cloacae*, and *E. coli*. Internal temperature more precisely predicted mortality compared to external temperature, indicating that a threshold of 85ºF (29.4ºC) was 86.0% predictive of mortality and 98.7% predictive of survival. Based on our findings, we recommend future studies involving BALB/c mice ESKAPEE pathogen infection use temperature monitoring as a humane endpoint threshold.

## Introduction

The increasing prevalence of multidrug-resistant (MDR) bacteria is a major threat to public health. Of particular concern are ESKAPEE pathogens (*Enterococcus faecium, Staphylococcus aureus*, *Klebsiella pneumoniae*, *Acinetobacter baumannii*, *Pseudomonas aeruginosa*, *Enterobacter cloacae*, and *Escherichia coli*), which are often nosocomial and can cause severe local and systemic infections [1]. Each year these bacteria cause over 35,000 deaths and over 1 million infections in the U.S [2], leading to significant morbidity and extended patient stays in hospitals. These outcomes are even more pronounced in immunocompromised or other susceptible populations, including those with hospital-acquired pneumonia (HAP) or ventilator-associated pneumonia (VAP), which can lead to increased susceptibility to lung infections by ESKAPEE bacteria. Complications in treatment have grown in recent decades due to bacteria becoming increasingly resistant to antibiotics [1], which is, in part, the reflection of limited new classes of antibiotics introduced to the market since the 1980s [3]. Although new antibiotics have been approved by the FDA during this time, they are mostly modified versions of previous classes, and resistance can emerge quickly because bacteria have already developed resistance mechanisms to the parent molecule [4,5]. Ongoing research into novel antibacterial therapies, including bacteriophages, vaccines, monoclonal antibodies, and other small molecules continues the search for the next new antimicrobial that will help alleviate this crisis [6–8]. These therapies represent many possible targets for future research and must be tested for preclinical safety and efficacy. A variety of *in vitro* and *in vivo* models have been developed using a tiered approach to assess efficacy against ESKAPEE pathogens [9], advancing only the most promising candidates.

Our previous research focused on the pathogenesis of ESKAPEE organisms via dermal wound or pulmonary inoculation and subsequent host physiology responses in preclinical murine models [10–13]. In the pulmonary model, immunosuppressed mice are inoculated intranasally to mimic cases of HAP/VAP, followed by treatment with different antibacterial candidates to evaluate to evaluate survival and the prevention of sepsis. The efficacy of the treatment is then measured clinically and histologically (e.g. weight change, clinical scoring, survival, bacterial burden). Recently, studies have suggested the pulmonary model should be standardized across multiple labs to allow comparisons between different antimicrobials and enable better prediction of efficacy [14].

Experimental protocols are designed with attention to using the appropriate number of animals, balancing the need for statistical relevance while supporting the principles of replacement, reduction, and refinement (also known as the “3Rs” alternatives). Nevertheless, to evaluate potential treatments at various dose-concentrations against multiple ESKAPEE species, hundreds to thousands of mice are often required to produce results with statistical significance. Unfortunately, computer models and other non-animal approaches cannot mimic the complex dynamics of the host such as: fluid movement, immune cell distribution, clearance, host response to bacterial infection, or downstream consequences to the host such as sepsis and death. Few survival models exist for ESKAPEE pathogens. This could be related to Institutional Animal Care and Use Committee (IACUC) limitations associated with U.S. Department of Agriculture (USDA) pain and distress Category E animal research protocols [14]. Non-steroidal anti-inflammatories (NSAID) [15,16] and opioid [17,18] analgesics can produce confounding immunomodulatory effects on mice and may impact bacterial pathogenesis. Therefore, proposals to investigate novel antimicrobials often receive IACUC approval to exclude the use of analgesics while the test article is evaluated, sometimes resulting in unrelieved pain in animals. Humane endpoints for euthanasia can be difficult to predict when relying on subjective evaluations of animal condition. Some protocols include a specific parameter, such as external body temperature, to assign a cut-off point to aid in euthanasia decision-making [19]. Many studies use a clinical scoring chart and/or combination of subjective and objective parameters to help identify animals requiring veterinary care or euthanasia prior to the end of the study [20–22]. These metrics are often based on clinically observable parameters, such as body weight, body surface temperature, internal body temperature, appearance, behaviour, hydration status, and respiratory effort. However, these parameters are not standardized across laboratory animal medicine [14].

In this study, we hypothesized that objective metrics could be established to determine humane endpoint criteria for early euthanasia in a pulmonary model of ESKAPEE pathogen inoculation for each individual pathogen in comparison to naïve animals. Our findings demonstrated that temperature could serve as an accurate predictor of mouse mortality during ESKAPEE pathogen infection.

## Materials and methods

### Mice and husbandry

Female BALB/c mice (*N* = 212) were purchased from Charles River (strain code 028). The mice used in these experiments were 6–10 weeks of age and weighed 15–22 g. All mice received irradiated food (PicoLab Rodent Diet 5053, LabDiet) and water *ad libitum*. All mice were housed in groups (*n* = 5) in sanitized solid-bottom individually ventilated cages (Sealsafe Plus GM500, Techniplast) on sterile paper bedding (Alpha-dri, Shepherd Specialty Paper) and were provided with environmental enrichment including a plastic Mouse Igloo with saucer wheel (BioServ) and a single Nestlet square (Ancare). Animals were maintained under a 12:12 hour light/dark cycle, with 10–15 air changes per hour, relative humidity of 30–70%, and room temperature between 68–79ºF (20–26.1ºC). From entry into the facility, all mice were housed under Animal Biosafety Level 2 (ABSL-2) conditions, where they acclimated to the facility for at least 5 days. Animals were identified using cage cards and tail marks with a permanent marker. Prior to the start of the study, all animals were monitored twice daily during routine animal health rounds.

### Murine pulmonary infection model

**Figure 1.**
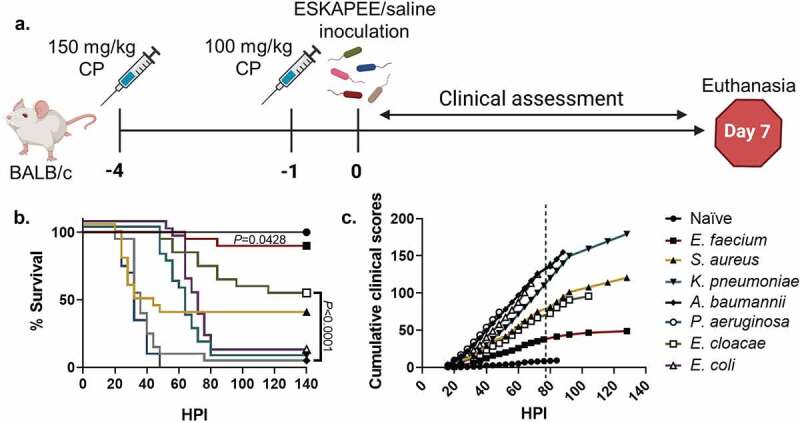
Study design, survival curve, and clinical assessment of ESKAPEE pathogens in BALB/c mice. **(a)** Animals were immunosuppressed with 150 mg/kg cyclophosphamide (CP) at Day−4 and 100 mg/kg at Day−1 and then inoculated with one of the ESKAPEE pathogens or sterile saline (Naïve). Clinical observations were made every 4 hours post-inoculation (HPI) throughout the assessment period. Animals were removed from the study if they were found dead in cage (FDIC). Surviving animals were euthanized at Day 7. **(b)** Percent survival by group. *P*-values represent the significance between mice inoculated with one of the ESKAPEE pathogens vs. the naïve group. For *E. faecium*, *P* = 0.0428. For the remaining pathogens, *P* < 0.0001. **(c)** Cumulative clinical scoring demonstrates disease progression over time. The dotted line represents euthanasia of naïve mice.

This study was performed in accordance with and under protocol 20-BRD-29, which was approved by the Institutional Animal Care and Use Committee (IACUC) at the Walter Reed Army Institute of Research (WRAIR). This model is similar to our previously described models of pulmonary infection [10,12,23]. A total of 20 mice (*n* = 20) were used for each bacterial species. Each group was randomly assigned to one pathogen or saline control (naïve), and every mouse received 150 mg/kg and then 100 mg/kg intraperitoneal (IP) injections of cyclophosphamide on Days −4 and −1 before inoculation (Figure 1a), respectively, a treatment which caused temporary neutropenia and allowed ESKAPEE pathogens to consistently establish infection. On Day 0, mice were anesthetized using 2.0–5.0% isoflurane gas in an induction chamber for approximately 5 minutes. Depth of anaesthesia was indicated by loss of consciousness and failure to react to noxious stimulus (i.e. toe pinch). Mice were then placed in sternal recumbency and an Implantable Programmable Temperature Transponder 300 (IPTT300) microchip with temperature reading capability (IPTT Pocket Scanner DAS-5007, Bio Medic Data Systems) was injected subcutaneously (SQ) into the dorsum approximately between the scapulae. Mice were then held in dorsal recumbency at a 45-degree angle with the head down, and 50 µL of ESKAPEE bacterial inoculum or sterile phosphate-buffered saline (PBS) was slowly infused into the nostrils using a pipet tip. Mice were returned to their cage in lateral recumbency and monitored until they recovered from anaesthesia as demonstrated by their ability to ambulate.

### Bacterial strains and inoculum preparation

The bacterial isolates and the colony forming unit (CFU) dose that were used in each respective inoculum for this study were selected based on the relevance of each strain to concurrent research efforts and/or its demonstrated virulence or antimicrobial resistance in clinical settings. The bacterial strains used were *Enterococcus faecium* strain 19434 (dosed at 5.7 × 10^6^ CFU), *Staphylococcus aureus* strain JE2 (dosed at 1.3 × 10^7^ CFU), *Klebsiella pneumoniae* strain KP4640 (dosed at 1.1 × 10^7^ CFU), *Acinetobacter baumannii* strain AB5075 (dosed at 1.5 × 10^7^ CFU), *Pseudomonas aeruginosa* strain PAO1 (dosed at 1.9 × 10^5^ CFU), *Enterobacter cloacae* strain 23391 (dosed at 1.2 × 10^7^ CFU), and an extra-intestinal pathogenic *Escherichia coli* (ExPEC) strain 15051 (dosed at 2.3 × 10^7^ CFU).

To prepare each inoculum for animal infection, 100 µL of an overnight culture was sub-cultured into 10 mL of fresh lysogeny broth (LB) in a 250-mL Erlenmeyer flask, grown at 37°C, and shaken at 250 rpm. Cells in the mid-exponential growth phase were harvested at an optical density in the range of 0.3–0.5 at 600 nm. Cells were washed three times with sterile PBS and then resuspended in PBS so that 50 µL of the suspension contained approximately 1.0 × 10^7^ CFU. The cell concentration of the suspension was verified via serial dilution and plating on LB agar.

### Animal observations

Beginning in the morning following inoculation, every animal was evaluated at 4-hour intervals. All evaluations were performed under a biosafety containment hood (Class II type A2, Labconco) and animals were handled using a tail hold to move them from their primary enclosure during evaluations. Internal body temperature was recorded using an IPTT300 microchip reader by placing the tip of the reader over the microchip injection site (dorsum between the scapulae) within approximately 8 cm of the animal. External body temperature was recorded using a non-contact infrared thermometer (JXB-178, Bercomm) at the midline of the abdomen, cranial to the most caudal teats, and within approximately 5 cm from the surface of the abdomen, while the animal was gently lifted by the tail and allowed to grasp onto the wire cage top. Qualitative clinical scores were recorded based on each animal’s general appearance, behaviour, hydration status, and respiratory effort (Table 1). Each animal was evaluated for every element of the four categories and assigned the point value associated with each individual observation. Therefore, an animal that demonstrated multiple components within a single category could receive cumulative points for every positive finding (i.e. a mouse that was vocalizing while ataxic would have received 4 + 6 = 10 points in the Behaviour category). Additionally, each animal was weighed every 12 hours at noon and midnight. The starting weight for each mouse was calculated as the average of the 3 weights taken on Days −4, −1, and Day 0. Weight loss percentage was calculated against that average starting weight and is represented as a change from 100% of the original weight. In one biological replicate of the *P. aeruginosa* group, the first weight post-inoculation was taken 4 hours earlier and the remaining assessment timepoints were identical.

**Table 1.** Clinical scoring point value assignment for selected clinical signs.

CATEGORY	DESCRIPTION	VALUE
Appearance	normal	0
	lack of grooming	1
	piloerection	1
	porphyrin staining	2
Behaviour	socially interactive	0
	hyperactive	1
	decreased mobility	1
	increased isolation	2
	vocalization	4
	ataxia	6
Hydration status	normal	0
	slightly dehydrated	1
	moderately dehydrated	3
	severely dehydrated	6
Respiration	normal pattern	0
	increased rate	1
	increased rate with abdominal component	3
	labored	6
	agonal	10

The primary endpoints in the study were either death or survival out to 7 days post-inoculation. Carcases of animals that succumbed to the test pathogen (as determined by rigour mortis) were deemed “found dead in cage” (FDIC) and were removed according to protocol. Animals that did not succumb to the pathogen of interest were humanely euthanized by carbon-dioxide asphyxiation followed by cervical dislocation.

To visualize trends and better predict mouse mortality, we used a previously demonstrated cumulative clinical scoring technique to show longitudinal assessment of disease progression and clinical outcomes per group over time [21]. Based on historical experience with these models, we selected a clinical score of 9 points as a subjective indicator of mortality and had used it as a humane endpoint threshold when considering early euthanasia. In this study, the protocol authorized animals to continue to the point of natural death whereas they would have otherwise likely been euthanized before that point. Therefore, animals that were FDIC continued to receive a score of at least 9 points at each subsequent timepoint until the end of the experiment. If an animal’s clinical score immediately prior to being FDIC was greater than 9 points, the animal continued to receive the higher score for the remainder of the experiment. The average clinical score within a group at a given timepoint was added to the previous average clinical score, reflecting the severity of disease over time (Figure 1).

### Bacterial enumeration and histological examination of infected tissues

In order to gather data regarding systemic bacterial burden in each model, an additional set of 8 groups of 4 mice were immunosuppressed with cyclophosphamide and then inoculated with one of the 7 ESKAPEE pathogens or sterile PBS in the same manner as previously described. At both 24 hours and 48 hours post-inoculation (HPI), 2 mice from each group were humanely euthanized by carbon-dioxide asphyxiation, and then organ tissue including the lungs, spleen, heart, liver, and kidneys from each animal was collected via gross dissection for subsequent histopathological evaluation. A portion of lung and splenic organ tissue was weighed and suspended in 1 mL of PBS and individually homogenized in a tissue grinder. Serial 10-fold saline dilutions of homogenates were cultured on lysogeny agar plates for bacterial enumeration and the number of bacterial colonies expressed as CFU per gram of organ. The remaining tissues were fixed in 10% buffered formalin for at least 21 days, embedded in paraffin, trimmed into 5 µm sections, stained with haematoxylin and eosin (H&E), and evaluated histopathologically by a board-certified veterinary pathologist who was blind to the treatments given to the various groups of mice. A minimum of 10 high-powered fields on each slide were evaluated for each specimen.

### Statistical analyses

For statistical power, the pulmonary infection experiment was split into two sets of five technical replicates (*n* = 5) and repeated once (*n* = 10) for one biological replicate (*n* = 20 total for each bacterial species). One set included two groups of five mice exposed to one of the following agents as previously described: *A. baumannii*, *E. coli*, *P. aeruginosa*, and sterile saline. The other set also included two groups of five mice exposed to one of the following agents as previously described: *E. faecium*, *E. cloacae*, *K. pneumoniae*, *S. aureus*, and sterile saline. GraphPad Prism version 9 was used to perform all statistical analyses. Statistical analyses were performed with *n* ≥ 19 BALB/c mice per inoculation group. Percent survival was analysed using Kaplan-Meier survival curves, and the Log-rank (Mantel-Cox) test was used to evaluate significance between non-infected groups and ESKAPEE inoculated groups. Mixed-effects analysis with Tukey’s and Dunnett’s multiple comparisons tests were utilized to evaluate statistical significance between naïve, survivors and FDIC mice for both internal and external temperature as well as percent weight loss data sets.

## Results

The purpose of this study was to evaluate a series of objective and subjective measures over the course of disease progression. It was anticipated that most animals would succumb to disease caused by the bacterial inoculation. The protocol was designed to allow animals to die naturally without intervention (USDA pain and distress category E), despite the animals’ anticipated deterioration, in order to better understand the clinical presentations and microbial pathogenesis of the ESKAPEE disease process. In this study, we wanted to evaluate temperature and weight loss as objective predictors of mortality. Overall, a total of 178 of the 180 mice from this portion of the study reached the anticipated endpoint. Two animals were euthanized prior to completion of the study due to animal welfare concerns (i.e. self-injury associated with the microchip implantation site). Here, we analysed the various clinical parameters caused by different bacterial species in BALB/c mice. We evaluated survival, internal temperatures, body surface temperature, combined clinical score, weight, and weight loss percentage for each group (Table 2). We argue that these parameters, temperature in particular, can be used to predict humane euthanasia which could ultimately reduce animal pain and distress.

**Table 2.** Clinical parameter measurements post-inoculation with ESKAPEE pathogens in comparison to naïve controls.

	Mice per group, n (FDIC^a^)	Survival time, HPI (median)	Internal temperature, ºF (median)	Body surface temperature, ºF (median)	Combined clinical score, points (median)	Weight, g (median)	Weight loss, % (median)
Control	39	–	80.1–105.4 (97.9)	86.5–97.8 (96.7)	0–8 (0)	12.5–21.3 (17.6)	−17–31 (6)
*E. faecium*	20 (2)	64–84 (74)	72.5–102.9 (97.2)	72.5–98.8 (96.6)	0–16 (2)	12.9–20.8 (16.2)	−3–27 (13)
vs. control			93.0–99.7 (96.8)	92.3–97.2 (96.6)	0–1 (0)	14.0–20.5 (17.4)	−1–19 (3)
prior to FDIC^e^			72.5–98.2 (85.4)	72.5–96.4 (84.5)	5–16 (10.5)	13.6–13. (13.7)	10–19 (15)
survivors^f^			94.6–98.8 (96.6)	96.4–97.1 (96.8)	1–2 (1.5)	13.8–17.9 (15.4)	6–27 (15)
*S. aureus*	20 (13)	20–48 (28)	72.7–100.9 (97.2)	75.0–98.1 (96.7)	0–10 (2)	11.5–21.2 (16.5)	−6–24 (13)
vs. control			94.6–101.1 (97.4)	96.1–97.7 (96.7)	0–0 (0)	13.2–21.3 (17.9)	−4–21 (5)
prior to FDIC			72.7–95.7 (79.5)	75.0–97.0 (91.0)	2–10 (7)	11.5–17. (15.1)	9–24 (13)
survivors			95.2–99.7 (96.3)	96.4–96.8 (96.6)	0–4 (3)	13.3–17.3 (15.3)	13–22 (17)
*K. pneumoniae*	20 (19)	48–80 (64)	67.6–99.7 (87.1)	72.0–99.1 (91.4)	1–15 (6)	12.3–20.4 (15.3)	−10–31 (19)
vs. control			94.6–101.5 (97.5)	96.4–97.1 (96.9)	0–8 (1)	12.5–19.4 (17.2)	1–23 (6)
prior to FDIC			67.6–93.4 (80.4)	72.0–96.3 (76.5)	6–14 (9)	12.3–16.3 (14.2)	−3–31 (23)
survivor			98.4	97.3	6	13.7	19
*A. baumannii*	20 (19)	20–76 (36)	69.3–102.0 (80.1)	68.2–97.7 (78.1)	0–2 (6)	13.8–20 (16.3)	8–24 (14)
vs. control			96.1–102.9 (98.8)	96.3–97.2 (96.7)	0–1 (0)	12.8–20.8 (17.5)	−1–24 (5)
prior to FDIC			70.3–84.9 (73.9)	68.2–92.2 (74.4)	4–13 (8)	13.8–17.7 (16.0)	9–24 (16)
survivor			100.4	96.8	1	15.3	12
*P. aeruginosa*	20 (20)	24–48 (32)	69.1–100.9 (78.1)	72.0–97.3 (75.7)	1–16 (6)	13.6–20.2 (16.8)	7–22 (14)
vs. control			96.6–103.5 (100.0)	96.6–97.4 (96.9)	0–1 (0)	12.8–20.8 (17.5)	−1–24 (5)
prior to FDIC			70.5–82.8 (73.6)	72.1–77.0 (74.4)	6–16 (9)	13.6–17.3 (15.9)	8–22 (16)
survivors			–	–	–	–	–
*E. cloacae*	20 (9)	48–116 (72)	83.1–100.4 (93.7)	64.1–97.2 (96.2)	0–12 (4)	10.8–21.1 (14.8)	5–35 (23)
vs. control			93.0–99.7 (96.8)	92.3–97.2 (96.6)	0–1 (0)	14–20.5 (17.4)	−1–19 (3)
prior to FDIC			85.1–96.8 (87.1)	90.1–96.7 (92.8)	4–9 (7)	10.8–15.3 (12.6)	22–35 (31)
survivors			96.6–103.5 (100.0)	96.2–96.8 (96.5)	2–4 (3)	12.7–15.7 (14.3)	22–29 (26)
*E. coli*	19 (18)	52–80 (68)	70.7–101.8 (89.2)	71.1–97.1 (89.8)	0–20 (7)	11.8–20.8 (14.8)	6–28 (20)
vs. control			91.4–100.2 (96.3)	93.7–97.0 (96.6)	0–3 (1)	14.0–20.5 (17.4)	−1–19 (3)
prior to FDIC			70.7–82.6 (76.0)	71.1–89.8 (74.0)	8–20 (13)	11.8–16.0 (14.0)	17–28 (24)
survivors			96.8	96.5	9	13.9	18

^a^HPI, hours post inoculation.

^b^FDIC, found dead in cage.

^c^analysis of values in all mice at all timepoints within the group.

^d^analysis of naïve mice at the median survival time for each pathogen.

^e^analysis of values in mice that succumbed to disease at the point immediately prior to the animal being “found dead in cage.”

^f^analysis of survivor mice at the median survival time for each pathogen.

### Survival and combined clinical scores were inversely related during ESKAPEE infection

Similar to other ESKAPEE pathogen studies, we evaluated survival as a primary endpoint. The strains used in these studies were either clinical isolates or established laboratory strains. Animals inoculated with *P. aeruginosa* succumbed to disease less than 48 HPI (0% survival) followed closely by mice inoculated with *A. baumannii* (5% survival) (Figure 1b). Most mice inoculated with *S. aureus* also rapidly succumbed to infection 32 HPI (35% survival) which was similar to previous studies (Figure 1b) [12]. Conversely, the *K. pneumoniae* (5% survival) and *E. coli* (5% survival) groups showed a delayed decline to morbidity starting at 60 HPI resulting in the majority of animals in each group succumbing to *K. pneumoniae* or *E. coli* by Day 4 (Figure 1b). While *E. cloacae* demonstrated a steady decline over the course of the week resulting in over 50% survival, the *E. faecium* group mostly survived to the experimental endpoint with 90% survival (Figure 1b). As detailed in the methods, cumulative clinical scores demonstrate longitudinal progression of disease severity. Therefore, when clinical scores increase, survival decreases (Figure 1c). Overall, the data demonstrated that clinical scores can be used to predict mortality.

### Weight loss during ESKAPEE infection was not reflective of mortality

**Figure 2.**
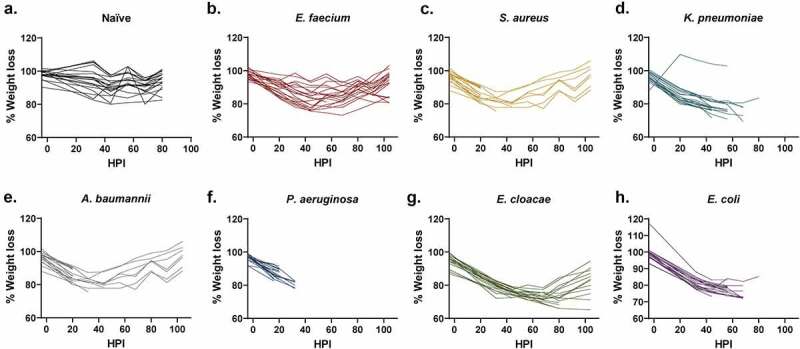
Assessment of weight loss vs. hours post-inoculation (HPI) with ESKAPEE pathogens in BALB/c mice. Each animal was weighed every 12 hours at noon and midnight. The starting weight for each mouse was calculated as the average of the 3 weights taken on Days−4, −1, and Day 0. Weight loss percentage was calculated against that average starting weight and is represented as a change from 100% of the original weight. Mixed-effects analysis with Dunnett’s multiple comparisons test was used to determine statistical significance. Compared against naïve mice, weight loss was statistically significant in all inoculated groups. **(a)** Naïve mice. **(b)** Mice inoculated with *E. faecium* (*P* < 0.0001). **(c)** Mice inoculated with *S. aureus* (*P* < 0.0001). **(d)** Mice inoculated with *K. pneumoniae* (*P* < 0.0001). **(e)** Mice inoculated with *A. baumannii* (*P* < 0.0001). **(f)** Mice inoculated with *P. aeruginosa* (*P* = 0.0021). **(g)** Mice inoculated with *E. cloacae* (*P* < 0.0001). **(h**) Mice inoculated with *E. coli* (*P* < 0.0001).

Weight loss is commonly used as a method to evaluate animal health [24, 25,26]. In fact, many protocols use ≥20% weight loss as a determinant for euthanasia. Historically in our work, we have used weight loss ≥30% as a euthanasia criteria threshold [10]. However, the method is time consuming and difficult to quantify as animals must be assessed against different starting weights. In this study, the majority of mice lost weight in the initial 24–48 HPI (Figure 2). Unfortunately, weight loss could not be correlated to mortality predictions. For example, the *E. cloacae* group showed both the greatest average and individual weight loss (23% and 35%, respectively) of any pathogen group (Figure 2g) but the moderate-to-severe weight loss in this group did not appear to coincide with high mortality (Figure 1). Interestingly, there were only a small number of mice across multiple pathogens that reached ≥30% weight loss yet still survived, which suggests that severe weight loss is generally indicative of a poor prognosis (Figure 2). Thus, weight loss is possibly a function of the systemic response of the animal to disease, such as the immune response, suggesting that animals may not have time to demonstrate ≥30% body weight loss before succumbing to the infection. Conversely, there were a substantial number of mice that were FDIC with only mild-to-moderate weight loss. For example, 8 of the 13 mice in the *S. aureus* were FDIC with weight loss ≤15%, including 4 mice with ≤10% weight loss (Figure 2c). Additionally, 8 of the 19 mice in the *A. baumannii* group were FDIC with weight loss ≤15% (Figure 2e). Finally, 10 of the 20 mice in the *P. aeruginosa* group were FDIC with weight loss ≤15%, including 4 mice with ≤10% weight loss (Figure 2f). Therefore, current percent weight loss may not function as a strong prognostic indicator for continued survival to the next observation time point. Furthermore, weight loss could be specific to the bacterial pathogen.

### Body temperature during ESKAPEE infection was predictive of mortality

Temperature is a common vital sign used to indicate animal health. Although there can be age-, sex-, breed-/strain-, and environmentally-dependent variations, a healthy mouse is typically able to thermoregulate at or around 99.5ºF (37.5ºC) [27]. Since temperature is an important clinical health indicator, we used multiple methods to better evaluate modalities of temperature recording in addition to their predictive value for mortality. Internal temperature was monitored using implanted microchips with a digital external reader and external temperature was measured using an infrared laser on the abdomen.

**Figure 3.**
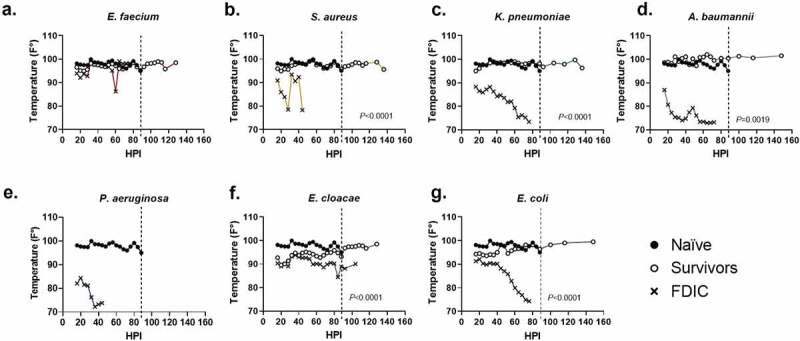
Assessment of internal temperature vs. hours post-inoculation (HPI) with ESKAPEE pathogens in BALB/c mice. ESKAPEE pathogen inoculated mice are presented in two groups: survivors and FDIC. A total of 19–20 mice (*n* ≥ 19) were used for each bacterial species (Table 2). Mixed-effects analysis was used to determine statistical significance. *P*-values represent the significance between mice that were survivors vs. FDIC for each ESKAPEE pathogen. The median temperature of each group and naïve animals is plotted over time. A black circle represents internal temperature for naïve mice. A white circle represents internal temperature for mice that ultimately survived. An X represents internal temperature for mice that were ultimately FDIC. Mice inoculated with **(a)** *E. faecium*, **(b)** *S. aureus*, **(d)** *K. pneumoniae*, **(e)** *A. baumannii*, **(e)** *P. aeruginosa*, **(f)** *E. cloacae*, **or (g**) *E. coli*. The dotted line represents euthanasia of naïve mice.

We measured internal temperature on a per bacteria basis and compared the naïve animals to inoculated animals that survived or were FDIC (Figure 3). In this study, temperature decrease correlated with mortality. *S. aureus*, *K. pneumoniae*, *A. baumannii*, *P. aeruginosa*, and *E. coli* inoculated mice demonstrated the greatest temperature decrement within the first 48 hours (Figure 3b–e,g) which corresponded to a decrease in survival (Figure 1). In contrast, mortality was less frequent in animals that were able to maintain thermoregulation closer to typical physiological parameters (Figure 3a,f).

When evaluating survivors, the overwhelming majority maintained an internal body temperature ≥90ºF (32.2ºC) throughout the entirety of the experiment (middle quartiles ranged from 95.9–98.8ºF (35.5–37.1ºC) with a standard deviation of 2.4ºF (1.3ºC)) (Supplemental Data 1), suggesting that the ability to thermoregulate is a strong predictor of survival (Figure 3). Across all mice that were FDIC, the median internal temperature was consistently much lower, and the median internal temperature immediately prior to FDIC was 75.2ºF (24.0ºC) (Table 2) with a standard deviation of 6.4ºF (3.6ºC). Internal temperature differences between survivor and FDIC mice were statistically significant for all inoculation groups except for *P. aeruginosa* (for which there were no survivors) and *E. faecium*. Interestingly, most groups (except *K. pneumoniae* and *E. faecium*) demonstrated a statistical difference between survivors and naïve mice as well, suggesting survivors were often clinically impacted by inoculation but were able to recover.

**Figure 4.**
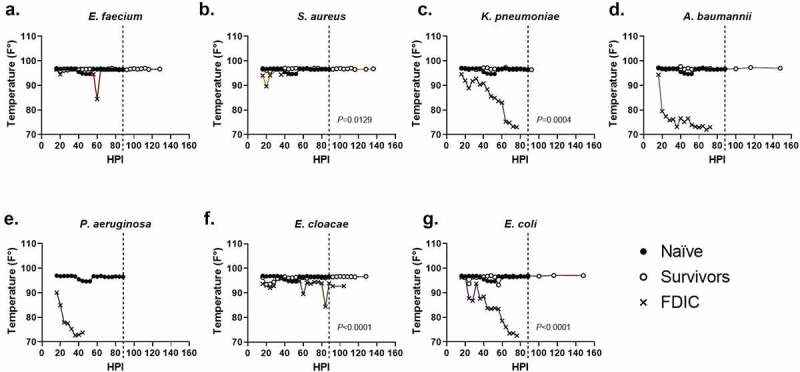
Assessment of external temperature vs. hours post-inoculation (HPI) with ESKAPEE pathogens in BALB/c mice. ESKAPEE pathogen inoculated mice are presented in two groups: survivors and FDIC. A total of 19–20 mice (*n* ≥ 19) were used for each bacterial species (Table 2). Mixed-effects analysis was used to determine statistical significance. *P*-values represent the significance between mice that were survivors vs. FDIC for each ESKAPEE pathogen. The median temperature of each group and naïve animals is plotted over time. A black circle represents external temperature for naïve mice. A white circle represents external temperature for mice that ultimately survived. An X represents external temperature for mice that were ultimately FDIC. Mice inoculated with **(a)** *E. faecium*, **(b)** *S. aureus*, **(d)** *K. pneumoniae*, **(e)** *A. baumannii*, **(e)** *P. aeruginosa*, **(f)** *E. cloacae*, **or (g**) *E. coli*. The dotted line represents euthanasia of naïve mice.

For external temperature evaluations, the majority of survivors maintained an external body temperature ≥90ºF (32.2ºC) throughout the entirety of the experiment (middle quartiles ranged from 96.5–96.8ºF (35.8–36.0ºC) with a standard deviation of 1.3ºF (0.7ºC)), suggesting similarly that the ability to thermoregulate is a strong predictor of survival (Figure 4). Across all mice that were FDIC, the median external temperature was consistently much lower, and the external temperature immediately prior to FDIC was 75.1ºF (23.9ºC) (Table 2) with a standard deviation of 8.6ºF (4.8ºC). External temperature differences between survivor and FDIC mice were statistically significant for *S. aureus*, *K. pneumoniae*, *E. cloacae*, and *E. coli*. Similar to internal temperature measurements, most groups (except *K. pneumoniae* and *E. cloacae*) demonstrated a statistical difference between survivors and naïve mice as well.

Overall, internal and external temperature measures were consistent and predictive of mortality. Additionally, a Δ_temperature_ comparison between the two recording methods (Δ_temperature_ = internal temperature – body surface temperature) at each individual time point for every reading revealed a median temperature difference of+1.2ºF (0.7ºC) higher internal temperatures (range of −16.5–15.5ºF (−9.2–8.6ºC)) with a standard deviation of 2.9ºF (1.6ºC), suggesting both methods are relatively comparable (Supplemental Data 1). According to the manufacturer, accuracy of the IPTT 300 implantable microchips is variable across temperature ranges but generally <1ºF (0.6ºC).

### CFU and histopathology revealed bacterial dissemination

CFU enumeration can demonstrate bacterial burden within pulmonary infection models [14]. Based on previous work with these models, bacteria colonize and begin infecting the lung tissue about 4 HPI [28]. At 24 HPI, the lung is infected (approximately 1.0 × 10^10^ CFU/g of lung tissue). Between Day 1 and Day 2, the bacteria begin to disseminate from the lung into the bloodstream (bacteraemia) and subsequently colonize and infect other organs. This systemic infection results in immune dysregulation and clinical signs of illness (sepsis). Between Day 2 and Day 3, the animals typically succumb to the infection and expire without intervention. In this study, we expected the largest bacterial burden in organ tissue to reflect the height of infection around 48 HPI for most of the ESKAPEE pathogens [22]. Since animals typically succumb earlier to *P. aeruginosa* infection, we measured bacterial burden at 24 HPI. Bacterial burden can be reflective of bacterial dissemination into the bloodstream, sepsis, organ dysfunction, and death. Excluding the *E. faecium* group, our results demonstrated a successful pulmonary inoculation in all groups (Table 3). All bacterial species (except for *E. faecium*) also displayed systemic pathology where inoculation yielded sufficient bacteraemia to result in substantial numbers of CFUs accumulating in splenic tissue (Table 3). Unfortunately, we cannot effectively distinguish bacterial burden in FDIC mice from post-mortem bacterial overgrowth. Therefore, while these bacterial burden data cannot confirm that non-survivors are dying as a direct result of sepsis, it does show that systemic exposure in this model is likely contributing to clinical observations which ultimately end in death.

**Table 3.** Colony forming units of ESKAPEE pathogens in pulmonary and splenic tissue.

	Mice per group, n	median CFU/g^a^ at 24 HPI^b^	Mice per group, n	median CFU/g at 48 HPI
Not inoculated				
lung	2	no growth	2	no growth
spleen	2	no growth	2	no growth
*E. faecium*				
lung	2	no growth	2	no growth
spleen	2	no growth	2	no growth
*S. aureus*				
lung	2	2.43E + 10	1	2.59E + 09
spleen	2^c^	5.56E + 04	1	no growth
*K. pneumoniae*				
lung	2	1.48E + 13	2	3.35E + 13
spleen	2	6.11E + 05	2	7.72E + 06
*A. baumannii*				
lung	2	1.33E + 13	1	3.13E + 13
spleen	2	5.18E + 08	1	9.17E + 06
*P. aeruginosa*				
lung	1	9.31E + 12	0	–^d^
spleen	1	2.00E + 08	0	–
*E. cloacae*				
lung	2	2.31E + 10	2	4.44E + 13
spleen	2	3.47E + 05	2	1.44E + 07
*E. coli*				
lung	2	1.82E + 13	2	3.01E + 13
spleen	2	1.67E + 06	2	9.28E + 06

^a^CFU/g, colony forming units per gram.

^b^HPI, hours post inoculation.

^c^no growth observed on one of the two samples.

^d^no mice survived to 48 HPI collection point.

**Figure 5.**
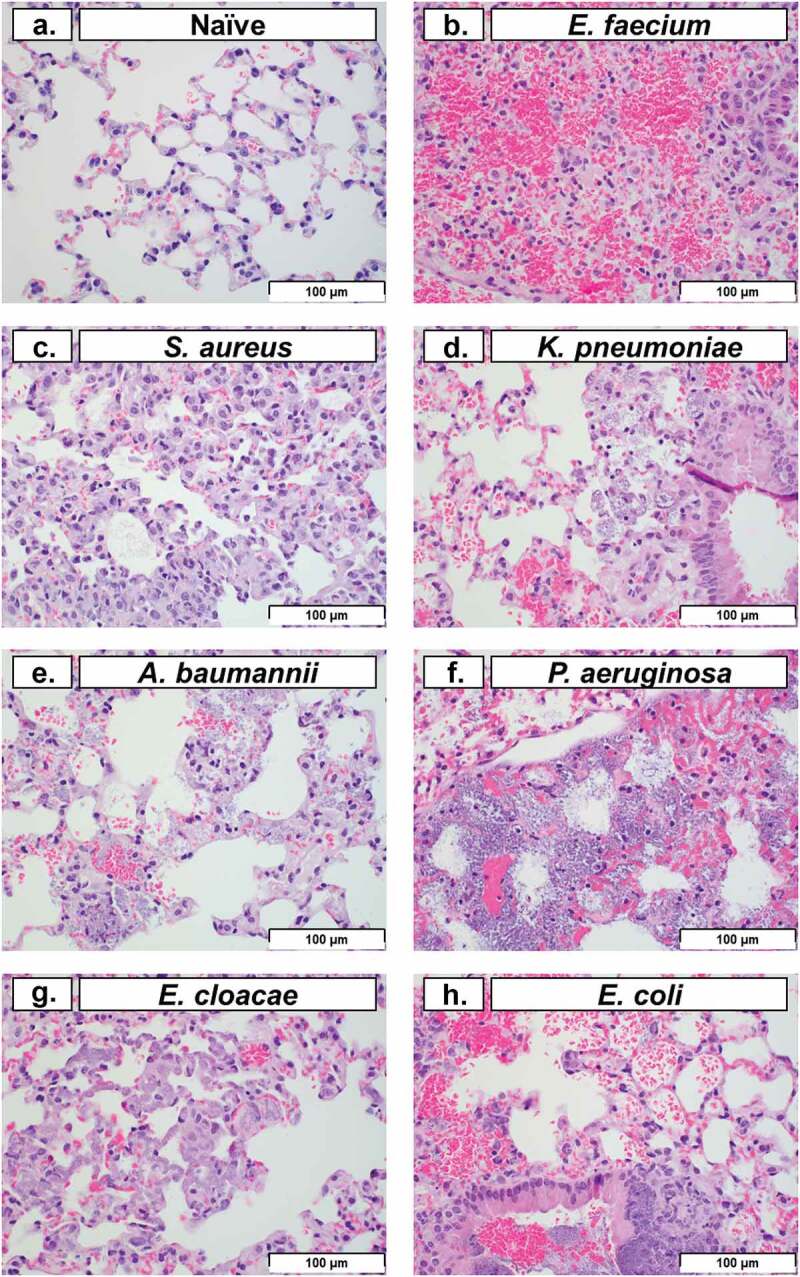
Histopathological analysis of pulmonary tissue post-inoculation with ESKAPEE pathogens in BALB/c mice. Hematoxylin and eosin (H&E) stained lung at 40× magnification. **(a)** Naïve, no abnormal findings. **(b)** *E. faecium*, alveolar septal necrosis, mild. **(c)** *S. aureus*, multifocal septal thickening, increased interstitial macrophages, increased alveolar macrophages. **(d)** *K. pneumoniae*, peribronchiolar and alveolar bacteria, septal necrosis, with septal and intrahistiocytic bacteria. **(e)** *A. baumannii*, septal necrosis, intrahistiocytic bacteria, extracellular bacteria in septa and alveoli. **(f)** *P. aeruginosa*, intracellular and extracellular bacteria, septal necrosis. **(g)** *E. cloacae*, alveolar septal necrosis, increased interstitial histiocytes, and intrahistiocytic bacteria. **(h**) *E. coli*, intrabronchiolar and alveolar extracellular bacteria, intrahistiocytic bacteria, septal necrosis, alveolar haemorrhage. Scale bars set at 100 µm.

Histopathology of lung tissue is a common method used to evaluate pulmonary tissue degradation caused by intranasal bacterial inoculation [29]. In this study, we performed histopathological analysis on lung tissue at 48 HPI (except for *P. aeruginosa*, which was performed at 24 HPI). Representative images of pulmonary tissue demonstrated typical post-inoculation results of each bacterium (Figure 5). Typically, neutrophils represent the largest leukocyte component of acute inflammation. However, cyclophosphamide treatment predictably caused significant neutropenia, which in turn obscured many of the classical signs of acute inflammation. Histopathological analysis of the lung in conjunction with clinical observations suggested that the haemorrhage (with fibrin and oedema present) was consistent with an acute process characterized by the increased number of alveolar macrophages and the lack of both hemosiderin and haematoidin (which might otherwise indicate a chronic process). In multiple images, macrophages with phagocytosed bacteria were observed (Figure 5), excluding post-mortem bacterial overgrowth post-inoculation. Histopathological findings in the spleen (data not shown), including bacteria in capillaries of the red pulp, provided evidence of systemic bacteraemia and dissemination from the lungs. In conjunction with the clinical presentation of the animals during the observation periods, spleen histopathology supported a clinical diagnosis of sepsis, organ colonization, and organ dysfunction as a potential reason for mortality with ESKAPEE pathogens. This is consistent with other studies that have evaluated similar observations in human patients, mice, and in other relevant animal models of infection [30–34].

## Discussion

From the work presented in this study, we demonstrated that temperature is a useful and objective indicator of mortality in ESKAPEE models of pulmonary infection in BALB/c mice. Comparing survivors against FDIC mice, we showed that internal temperature differences were statistically significant for *S. aureus*, *K. pneumoniae*, *A. baumannii*, *E. cloacae*, and *E. coli* (Figure 3) and external temperature differences were statistically significant for *S. aureus*, *K. pneumoniae*, *E. cloacae*, and *E. coli* (Figure 4). However, internal temperature was the more accurate measure (Figure 3), with less variability and more consistency across almost all the bacterial pathogens. Our findings were consistent with multiple previous studies demonstrating that both external and internal temperature measurements were important in predicting mortality in mice infected with various infectious pathogens. Mei et al. demonstrated similar findings between internal and external temperature measurement methods in a lipopolysaccharide (LPS) endotoxin-induced hypothermia model [35], and our study builds on this work in active infection models for all the ESKAPEE pathogens. Initial increases in temperature were seen within the first 20–40 hours, which could be linked to ESKAPEE pathogen exposure to the immune system and part of the response to pathogen-associated molecular patterns (PAMPs), damage-associated molecular patterns (DAMPs), and chromatin-associated molecular patterns (CAMPs), as host cells become lysed, and inflammation increases [36–38]. However, a collapse in body temperature is then observed, which can correlate with dissemination and mortality (Figure 3, 4).

In order to inform clinical decision-making based on the results of this study, temperature thresholds can be defined to drive humane endpoint algorithms. For example, if we had used an internal temperature threshold of ≤90ºF (32.2ºC) as a humane endpoint in this study, we would have accurately predicted mortality in 99 out of 100 (99.0%) animals that were ultimately FDIC and survival of 68 out of 78 (87.2%) animals (Supplemental Data 1). With a threshold of ≤87.5ºF (30.8ºC), we would have accurately predicted mortality in 94 out of 100 (94.0%) animals that were ultimately FDIC and survival of 74 out of 78 (94.9%) animals. With a lower threshold of ≤85ºF (29.4ºC), we would have accurately predicted mortality in 86 out of 100 (86.0%) animals that were ultimately FDIC and survival of 77 out of 78 (98.7%) animals. At the ≤85ºF (29.4ºC) threshold, the majority of false negative predictors of mortality came from the *E. cloacae* (6 out of 14), *S. aureus* (4 out of 14), and *K. pneumoniae* (4 out of 14) groups (Supplemental Data 1). These findings suggest that an internal temperature cut-off threshold of 85ºF (29.4ºC) is a useful predictor of mortality with both sensitivity and specificity for *E. faecium*, *A. baumannii*, *P. aeruginosa*, and *E. coli*.

Similarly, for external temperature, if we had used a threshold of ≤90ºF (32.2ºC) as a humane endpoint in this study, we would have accurately predicted mortality in 77 out of 100 (77.0%) animals that were ultimately FDIC and survival of 69 out of 78 (88.5%) animals (Supplemental Data 1). With a threshold of ≤87.5ºF (30.8ºC), we would have accurately predicted mortality in 84 out of 100 (84.0%) animals that were ultimately FDIC and survival of 75 out of 78 (96.2%) animals. With a threshold of ≤85ºF (29.4ºC), we would have accurately predicted mortality in 74 out of 100 (74.0%) animals that were ultimately FDIC and survival of 78 out of 78 (100%) animals. At the ≤85ºF (29.4ºC) threshold, the majority of false negative predictors of mortality came from the *S. aureus* (11 out of 26) and *E. cloacae* (7 out of 26) groups (Supplemental Data 1).

These findings, in addition to a wider degree of variation among external temperature readings compared to those of internal temperature, demonstrate that internal temperature monitoring is a stronger predictor of mortality and has a smaller number of false negatives across the different ESKAPEE pathogens. While an external temperature laser can “miss” the target (i.e. readings from a distal limb or an area of the body that has thicker hair), microchips provide a temperature from inside the body which yields a closer estimate of core body temperature. For single use study design, the microchip can be disposed of with the animal carcase or incinerated with other biomedical waste. However, the IPTT-300 chips are reusable (according to the manufacturer) if the glass casing remains intact, which can be achieved by recovering the chip after euthanasia followed by disinfection and re-sterilization. At an average cost of over $10 per chip, re-use makes them a potentially cost-effective means for larger studies [39]. Because microchip placement causes momentary pain in mice that are awake, microchip placement under anaesthesia (as performed in this study) should be considered as a protocol refinement to maximize animal welfare. Additionally, microchips do not require significant restraint or result in mucosal tearing as compared to traditional methods such as rectal temperature probes [40]. In our future work, we plan to use temperature monitoring in addition to cumulative clinical scoring as another parameter for decisions related to animal monitoring frequency as well as humane euthanasia criteria. Although internal temperature had better sensitivity and specificity for mortality than external temperature as an individual parameter, external temperature measurement may still be a valuable tool for future studies when designing clinical scoring parameters.

Historically, we had estimated that a clinical score of 3–8 suggested an animal was experiencing mild pathology related to infection and required increased monitoring frequency. Additionally, a score of 9 or higher represented severe disease burden and indicated that euthanasia should be considered. In this study, we demonstrated that a score of ≥ 9 points is a high specificity metric that can predict mortality without pre-emptively euthanizing significant numbers of healthy mice. This is particularly true with *E. faecium*, *K. pneumoniae*, *E. cloacae*, and *E. coli*, which took longer to establish an infection. However, clinical scoring remains highly subjective and likely includes variation within and between personnel. While these findings may be useful for pulmonary ESKAPEE pathogen models in BALB/c mice, there are considerations such as mouse strain and bacterial strain differences that will need to be evaluated in order to standardize a mouse pneumonia model [14].

In this study, if we had used a combined clinical score threshold of ≥ 9 points as a humane endpoint, we would have accurately predicted mortality in 71 out of 100 (71.0%) animals that were ultimately FDIC and survival of 75 out of 78 (96.2%) animals. This highlights further that internal temperature measurements serve as a better mortality and survival predictor, at 86.0% and 98.7%, respectively. With a lower threshold of ≥ 8 points, we would have accurately predicted mortality in 83 out of 100 (71.0%) animals that were ultimately FDIC and survival of 65 out of 78 (83.3%) animals. At the ≥9 points threshold, the majority of the false negative predictors of mortality came from the *S. aureus* (10 out of 29), *A. baumannii* (9 out of 29), and *P. aeruginosa* (7 out of 29) groups. These false negatives could be indicative of the subjective nature of clinical assessments of appearance, behaviour, hydration, and respiration scoring. These findings suggest that a combined clinical score cut-off threshold of ≥ 9 points is a useful predictor of mortality with sensitivity and specificity for *E. faecium*, *K. pneumoniae*, *E. cloacae*, and *E. coli*.

Surprisingly, weight loss did not represent a useful metric for this model. As previously stated, this is a common parameter used in many studies and our own protocols. However, it appears that there are several pathogen species, especially *S. aureus*, *A. baumannii*, and *P. aeruginosa*, which cause death so rapidly that extreme changes in weight are not observed. We suspect that this is related to the quick onset of sepsis and a toxic-shock-like syndrome which results in rapid death. Conversely, one pathogen species, *E. cloacae*, resulted in several examples of extreme weight loss (≥30%), yet death was not observed (Figure 2). For these reasons, weight loss is neither sensitive nor specific as a parameter for mortality predictions in this model.

We have identified some limitations regarding our data acquisition approach. Distance-to-spot ratio of the laser thermometer, which is a measure of accuracy and precision of the device, may vary between device type and manufacturer. Future studies may wish to assess this parameter to improve the rigour of the findings. Additionally, novel temperature acquisition technologies and more advanced data collection approaches may be useful to provide robust telemetric data collection at more frequent time points or even continuously with fewer direct handling events throughout the study. These technologies may reduce the wide range of internal and external temperatures observed immediately prior to FDIC, which could provide a more clearly defined temperature threshold for euthanasia decision-making for each infectious agent. However, this point further emphasizes the importance of using multiple parameters for euthanasia decision-making, as no individual factor is universally predictive of clinical outcomes.

Another aspect to consider is mouse strain selection. In our study we use BALB/c mice for a pulmonary infection model. However, C57/B6 and CD-1 outbred mice are also standards for the antibacterial community [14], and treatments that work in these mouse strains serve as important preclinical data to move into larger animal species and eventually people. Notably, there are substantial differences between the mouse strains that would need to be evaluated individually before these objective and subjective clinical indicators could be extrapolated beyond BALB/c mice. The use of other immunologically more susceptible mouse strains could reduce or eliminate the need to immunosuppress the animals with cyclophosphamide [41,42]. Alternatively, aged mice or genetically modified mice with impaired innate immune responses could also aid in the reduction of cyclophosphamide usage [43–45]. Not only would this lead to a potential animal refinement (fewer injections and fewer aversive handling experiences before the study) but can also reduce occupational exposure risks associated with such chemical agents [46]. Furthermore, mouse strains with an intact immune system could more precisely depict bacteria-induced pathology that is not captured in this experimental design. Also, while we try to reduce the amount of distress related to handling the mice, we question the impact of such frequent handling (every 4 hours) on survival, clinical conditions, and other parameters that we observed [47].

Despite these factors, our study has provided valuable parameters to predict mortality and improve animal welfare. In future work, additional elements can further improve these findings. It will be important to include both male and female mice in our studies to fully understand the different presentations related to hormonal variation between the sexes during infection [48,49]. In addition to validating this model in other mouse strains and organ/system models, we are interested in evaluating the relationship between bacterial burden and morbidity/mortality in the mice. Additional future analysis of clinical scoring sub-components (Supplemental Data 1) may be warranted to identify whether certain sub-components represent an outsized influence on mortality predictions. Future studies could also include more frequent CFU collection time points in addition to robust clinical pathology (i.e. complete blood count, chemistry panels, and necrosis specific immunohistochemistry markers) before inoculation, during infection, and immediately prior to final tissue collection/euthanasia. We also observed anecdotal evidence that there may be substantial temperature variation based on the total number of mice that remain in the cage throughout the study and how much time they spend huddled together in a nest or in the igloo. We cannot predict exactly which mice will develop severe infections post-inoculation and how many will succumb to the disease, but we are curious to understand whether variable numbers of mice throughout the experiment impacts mortality of the remaining conspecifics in that group.

The data presented above show predictive endpoints for each ESKAPEE bacterium in BALB/c mice. Therefore, in support of the principles of the 3Rs and ethical animal research, we recommend that laboratories that use these types of models should develop humane endpoint thresholds to include temperature monitoring of mice. Herein, we demonstrated that temperature is an effective objective parameter for establishing humane endpoints. Importantly, pilot studies are warranted in similar work to establish precise thresholds, justifying the continued use of these animals by avoiding unnecessary pain and distress and maximizing ethical and humane considerations. Subjective parameters, such as well-designed clinical scoring charts, can also be useful when making humane endpoint decisions. Even when mortality and clinical parameters are not correlated, a pilot study that evaluates these metrics can be useful in establishing thresholds for that specific study model. In such instances, USDA Category E animal research protocols which involve unalleviated pain or distress will require a scientific justification to gain IACUC approval. Therefore, pilot study findings can demonstrate that replacements, refinements, and reductions were thoroughly evaluated during protocol development and reliable alternatives are not available.

In this study, we determined that refinements to the objective euthanasia criteria can be established to alleviate unnecessary pain and distress in study animals. Temperature monitoring, specifically internal microchips, can provide consistent early indicators of impending death and inform euthanasia decision-making algorithms. Based on the large numbers of mice that are routinely required for antimicrobial development, we estimated that successfully achieving just a 12-hour pre-emptive euthanasia in merely 10% of all mice on our protocol would equate to over one entire mouse-year’s-worth of pain and distress being eliminated from the study. However, this data suggests that the impact of this work may improve animal welfare to an even more significant degree. Based on our findings, we will continue to use temperature as an objective parameter to evaluate animal conditions and make decisions regarding humane euthanasia.

## Supplementary Material

Supplemental Material

## Data Availability

The authors confirm that the study data is available within the article and supplemental materials (openly available in figshare at https://doi.org/10.6084/m9.figshare.22257727.v1).
